# Reliability of the In Silico Prediction Approach to In Vitro Evaluation of Bacterial Toxicity

**DOI:** 10.3390/s22176557

**Published:** 2022-08-31

**Authors:** Sung-Yoon Ahn, Mira Kim, Ji-Eun Bae, Iel-Soo Bang, Sang-Woong Lee

**Affiliations:** 1 Pattern Recognition and Machine Learning Lab, Department of AI Software, Gachon University, Seongnam 13557, Korea; 2 Department of Microbiology and Immunology, Chosun University School of Dentistry, Gwangju 61452, Korea

**Keywords:** protein, toxin, virulence factors, BERT

## Abstract

Several pathogens that spread through the air are highly contagious, and related infectious diseases are more easily transmitted through airborne transmission under indoor conditions, as observed during the COVID-19 pandemic. Indoor air contaminated by microorganisms, including viruses, bacteria, and fungi, or by derived pathogenic substances, can endanger human health. Thus, identifying and analyzing the potential pathogens residing in the air are crucial to preventing disease and maintaining indoor air quality. Here, we applied deep learning technology to analyze and predict the toxicity of bacteria in indoor air. We trained the ProtBert model on toxic bacterial and virulence factor proteins and applied them to predict the potential toxicity of some bacterial species by analyzing their protein sequences. The results reflect the results of the in vitro analysis of their toxicity in human cells. The in silico-based simulation and the obtained results demonstrated that it is plausible to find possible toxic sequences in unknown protein sequences.

## 1. Introduction

Humans spend over 90 percent of their time indoors, whether at home or at work [1]. Upon entering any indoor space, the air can instantly be contaminated with pathogens that may have traveled inside, whether through an open door or from a possible host carrying a disease. Contaminated air drastically increases the chance of infection via airborne transmission. Prevalent diseases, such as tuberculosis, asthma, and COVID-19, pose serious health risks as they sometimes lead to the death of their hosts. The World Health Organization (WHO) announced that an average of 3.8 million people lose their lives every year because of contaminated indoor air [2].

Bacteria and fungi are the most common airborne pathogens. These organisms have specific ecological niches and can adapt to the environment. Living organisms produce proteins derived from a chain of peptides, which act as building blocks in many organisms. The roles of proteins include the creation of hormones that affect various parts of the organism for different purposes, such as reproduction and heart rate control. Proteins also participate in chemical reactions within an organism as catalysts in the form of enzymes responsible for speeding metabolism [3]. Although proteins perform many valuable functions, they sometimes harm other organisms. These proteins are known as toxic proteins or toxins. These toxins act as virulence factors and cause diseases [4,5].

Due to globalization and advancements in transportation, the number of people moving from one place to another, often from country to country, is steadily increasing. These factors facilitate the spread of highly contagious and deadly diseases. Diseases often produce unpredictable outcomes, including spontaneous mutations, in which some variants become more contagious or deadly [6,7]. Therefore, it is crucial to understand mutations and predict the toxicity of a particular variant to create countermeasures. However, traditional in vivo and in vitro methods are time-consuming and expensive. In silico methods, however, provide faster results. Although they may not be sufficiently accurate, they can help guide researchers in identifying toxic sequences.

Studies in bioinformatics have revealed that incorporating deep-learning techniques to analyze genome and amino acid sequence data is often helpful in many subtasks. For instance, finding DNA-protein-interacting areas using reinforcement learning [8] and predicting the 3D structure of a protein [9] have significantly decreased the workload of many microbial studies. In addition, various methods are being employed to predict protein toxicity. Traditional machine learning methods, such as support vector machines (SVMs) and random forests (RFs), have been used for ToxinPred [10]. Clantox uses boosted stump classifiers to classify toxic and nontoxic animal proteins. Deep learning techniques have also been used to predict toxic protein sequences [11]. For instance, TOXIFY embeds toxic protein sequences using the Atchely factor matrix and runs it through a set of GRUs [12]. ToxDL combines protein domain knowledge with features derived from a CNN module for prediction [13]. ToxIBTL uses FEGS and the BLOSUM62 matrix to embed protein sequences, merge both features, and pass them through an information bottleneck layer [14].

Language models developed for natural language processing have yielded promising results over the past few years. The transformer model suggested by Vaswani has outperformed the previous state-of-the-art models, with fewer required computations and higher bilingual evaluation understudy (BLEU) scores [15]. Newer and better transformer-based models, such as bidirectional encoder representations from transformers (BERT) [16], have proven that pretrained language models improve the performance of many natural language tasks, and these models have been used to solve other problems such as image classification and semantic segmentation [17]. ProtBert is one of many target-specific BERT models. As suggested by Elnaggar, it has more computation layers than the original BERT implementation and is pretrained using protein sequences from UniRef and BFD [18].

In this study, we propose the use of a fine-tuned ProtBert model to predict bacterial proteins that may act as virulence factors. We first tested the model on existing toxic protein datasets to determine whether it could outperform previous methods for toxic protein classification. We then trained the model on a new dataset, where we labeled virulence factors as toxic protein sequences, to determine whether the toxic-protein-prediction performance would improve when compared to using only toxic protein sequences for training. Finally, we applied the model to random protein sequences of four common bacteria found in indoor conditions.

## 2. Materials and Methods

### 2.1. Benchmark Dataset

Although our study aimed to uncover potential toxic proteins of bacteria, we tested our method to compare it with other existing toxic-protein-prediction models to determine the validity of our method. Three independent datasets were used to evaluate the performance of the proposed method. The first dataset consisting of known animal toxins was collected from a previous study [11]. It consists of 4472 positive and 6341 negative samples from UniProt’s Animal Toxin Annotation Project [19]. All toxic proteins in the datasets were labeled as positive, and nontoxic proteins as negative. To differentiate it from other datasets used in this study, the first dataset was named the toxDL dataset.

The second dataset was collected from another study [20] to predict toxic proteins of known bacterial species. This dataset consists of 183 positive samples and 382 negative samples, and was named the BTXpred dataset.

For the final dataset, we combined two separate datasets, each containing different types of protein sequences. The VFDB [21] dataset, which consists of 25,288 bacterial proteins labeled as virulence factors, was used to make up the positive samples. We wanted to determine whether the model trained on protein sequences that had more information on hazardous factors would provide better predictions for finding potential toxic proteins. For the negative sample data, all 17,821 nontoxin samples from toxinpred2 [22] were used. The newly created third dataset is hereafter referred to as the combined dataset.

For an unbiased evaluation of our model on toxic bacterial proteins, we downloaded 18,194 bacteria from SwissProt [23], from which 373 proteins are labeled for toxin activity. We excluded 127 proteins that overlapped with the BTXpred dataset as it also collected data from [23]. In total, 216 proteins were used as positive samples. From the remaining 17,821 bacteria proteins that were not labeled with toxin activity, we randomly selected 1000 samples and used them as negative samples. The information of the dataset is listed below in Table 1.

In the toxDL dataset, proteins composed of more than 1002 amino acid residues were truncated for implementation [13]. However, truncating protein sequences may cause the model to learn mislabeled protein sequences, as altering or truncating a protein sequence may alter protein function and thus mislead prediction analysis. Therefore, proteins having less than or equal to 1002 amino acids were chosen for the third and test datasets. For the BTXpred dataset, the length of its sequences was relatively shorter than that of the other datasets and did not trigger any issues when training the model. Consequently, all data used for training in this study were obtained from natural protein sequences.

### 2.2. MTT Cell Viability Assay

To verify whether our method can find potential toxicity-related sequences in unlabeled protein sequences, we chose four well-known bacterial species that are commonly found in indoor air. The selected species were *Staphylococcus aureus*, *Staphylococcus epidermidis*, *Bacillus subtilis*, and *Micrococcus luteus*. All bacteria were heat-inactivated before cytotoxicity assay. The MTT [3-(4,5-dimethylthiazol-2-yl)-2,5-diphenyltetrazolium bromide] assay, a representative method for measuring cellular metabolic activity and evaluating cytotoxicity caused by toxic agents [24], was employed to assess bacterial toxicity in human cell lines including MRC5 (human lung fibroblast cell) [25], HeLa (human epithelial cell) [26] and YD38 (human oral squamous cell) [27], which were purchased from the Korean Cell Line Bank. The cells were cultured in Minimum Essential Medium Eagle (MEM, WELGENE) or Roswell Park Memorial Institute (RPMI 1640, WELGENE) media supplemented with 10% FBS and 1% penicillin-streptomycin solution (WELGENE).

Human cells were seeded into a 96-well plate (SPL) at a density of 2 × 10⁴ cells per well (MRC5 and YD38) or 1 × 10⁴ cells per well (Hela), and cultured in a CO_2_ incubator with 5% CO_2_ at 37 °C. After 24 h, serially diluted bacterial samples were added to the adherent cells. MTT assay was performed after incubating mixtures for 24 h, then the optical density of the wells was determined using an ELISA plate reader at 580 nm.

### 2.3. Collection of Unknown Protein Sequences

**Figure 1:**
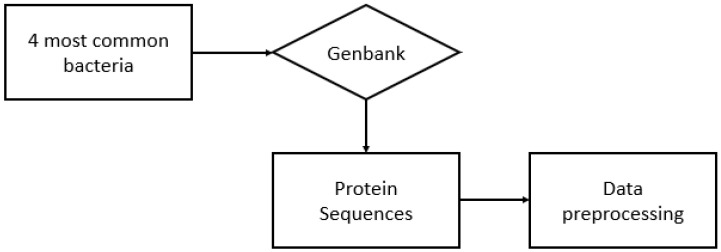
Pipeline for unknown protein data collection.

To collect the protein sequences of all four species, we searched GenBank [28] and selected the protein sequences. To find species-specific traits for toxic proteins, all proteins labeled “MULTISPECIES” were excluded, as some species may share certain toxic proteins with their neighboring variants but not trigger a toxic effect. In addition, any protein with an already labeled function was excluded. Figure 1 shows the pipeline of our data-collection scheme. Table 2 shows the number of protein sequences collected for each species.

### 2.4. Classification Model for Protein Sequences

The Hugging Face [29] implementation of ProtBert by Mani was used along with an additional classification layer for training the model [30]. As their implementation was intended for discriminating subcellular information of the input protein sequences, we made some adjustments by changing the number of classes. The classification layer consisted of a dropout layer to reduce overfitting and a fully connected layer to decrease the dimension size, followed by a hyperbolic tangent (tanh) function for classification. 

The equation for tanh is as follows:

(1) $$\tanh\left(x\right)=\frac{2}{1+e^{-2x}}-1$$

where *x* is the feature vector produced in the previous layer, and the prediction results of tanh range from −1 to 1. A positive number indicates a toxic protein and a negative number indicates the absence of toxicity.

To maximize the performance of the model, each dataset was pretrained for 20 epochs. After choosing the second lowest loss within the pretrained results, the model was fine-tuned using different learning rates and dropouts for the ToxDL data, BTXpred data, and combined data.

### 2.5. Evaluation Metrics

From Table 1, it can be observed that the distribution between the positive and negative samples differs significantly. Therefore, the evaluation metrics used for validation and testing did not include accuracy, which may not be objective. Instead, we used the F1 score, Matthews correlation coefficient (MCC), area under the precision–recall curve (AUPRC), and area under the receiver operating characteristic curve (AUROC). The metrics used for the evaluation were as follows: TP represents true positives, indicating toxic proteins correctly classified as toxic proteins. TN stands for true negative and represents nontoxic proteins correctly predicted as nontoxic proteins.

1. *F*1 *score*:

(2) $$F1\;score=1+\frac{2TP}{FP+FN}$$

2. *MCC*:

(3) $$MCC=\frac{TP\times TN-FP\times FN}{\sqrt{\left(TP+FP\right)\times \left(TP+FN\right)\times \left(TN+FP\right)\times \left(TN+FN\right)}}$$

## 3. Results

### 3.1. In Vitro Cellular Toxicity Test

**Figure 2:**
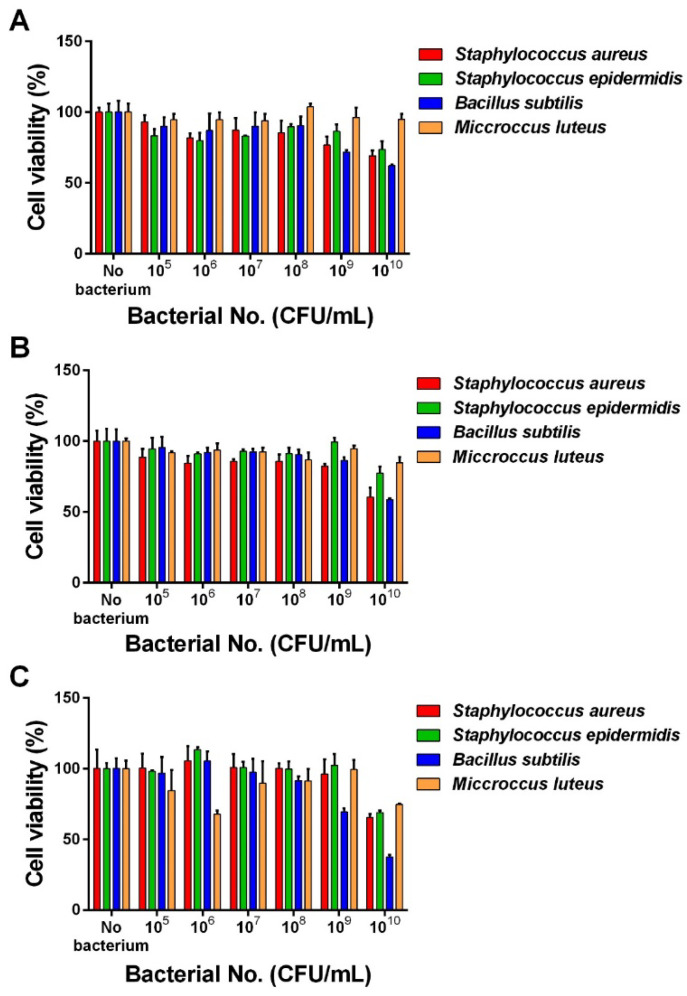
In vitro evaluation of bacterial toxicity in human cells. Effects of four species of bacteria on the viability of MRC5 (**A**), HeLa (**B**), and YD38 (**C**) cells analyzed by the MTT assay 24 h after the bacterial samples were added to human cells pre-seeded in 96-well plates. Results are expressed as cell viability as a percentage of cells incubated without bacteria. Experiments were repeated twice, with each condition being assessed in triplicates. Data are shown as the mean ± SD. CFU; colony forming unit.

To test the reliability of our in silico prediction program, we investigated the effects of four bacterial species on cell viabilities of MRC5, HeLa, and YD38 cells. Results showed cytotoxic effects of bacterial samples on all human cell lines used in this study, except *M. luteus* on MRC5 and HeLa cells, and their cytotoxic effects were increased in proportion to bacterial burden. The results can be viewed in Figure 2. At the highest concentration of bacteria (10^10^ CFU/mL), *S. aureus*, *S. epidermidis*, and *B. subtilis* reduced cell viabilities to 69%, 73%, 62% in MRC5 cells, 60%, 77%, 58% in HeLa cells, and 66%, 65%, 33% in YD 38 cells, respectively. *M. luteus* was cytotoxic only to YD38 cells. This result demonstrates that bacterial contents from these bacterial species can be cytotoxic in a dose- and cell type- dependent manner, proposing a considerable method to evaluate in vitro cellular toxicity of indoor air bacteria.

### 3.2. Comparison of Performance with Previous Toxic Protein Classification Methods and Datasets

Table 3 shows the results of the proposed method and other previous methods trained on the toxic animal protein dataset. The prediction results for BLAST [30], BLAST score [31], InterProScan [32], Hmmsearch [33], Clantox [11], toxinpred [10], and ToxDL [13] were collected from the results published in [13]. The results of ToxIBTL were obtained from results published in [14]. For ToxDL [13] and ToxIBTL [14], the authors of each publication measured the average of the results after ten experiments. The highest scores in each column are highlighted in bold. It can be seen in Table 3 that our method achieves the performance of the current state-of-the-art model, with only a small gap between the F1 and MCC scores.

Table 4 shows the prediction results from BTXpred [20] and our method for the second experiment on the toxic bacterial protein dataset. In BTXpred [20], five-fold cross-validation was used to test their approach. To accurately compare the performance of our method, we used the same validation technique. We compared our method using accuracy and MCC because these metrics overlapped with our evaluation metrics. Table 4 shows that our method outperformed BTXpred by +1.91% in accuracy and +0.0141 in MCC. In addition, we also trained the TOXIFY model [12] using the BTXpred dataset; however, we were not able to conduct a five-fold cross-validation and used its own accuracy score. Its training result is also listed in Table 4.

**Figure 3:**
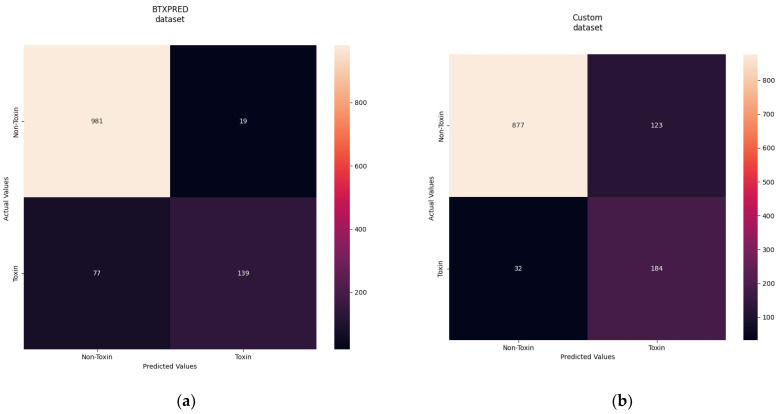
(**a**) Confusion matrix on the test data using the model trained using BTXpred data; (**b**) confusion matrix on the test data using the model trained using the combined VFDB and toxinpred2 data.

The results of the final experiment, in which we trained the model on the combined dataset [21,22], are presented in Table 5. Here, we achieved high scores in all metrics, with an F1 score of 0.953, MCC of 0.886, auROC of 0.956, and auPRC of 0.940. The results of the second experiment were better than those of the first experiment, which was conducted on toxic animal proteins. As bacterial proteins are 30–40% shorter than animal proteins [34], it is easier for the model to identify distinctive features between amino acids. Table 6 presents the performance results of our method using the test data. For the classification of toxic bacterial proteins, the model trained using BTXpred showed better results, with +0.0754 in F1, +0.1205 in MCC, and +0.1013 in auPRC. Figure 3 shows the confusion matrix for the two test results. Both models had a high TP rate, and most toxic proteins were correctly classified as toxic. Although the model trained using BTXpred data detected fewer toxic proteins, we believe that this was due to data unavailability. Thus, the model could not entirely learn all the features of toxic bacterial proteins. However, the model trained using the combined dataset had a higher TP rate and only misclassified 32 proteins as nontoxic. Nevertheless, the model trained on the combined dataset had a higher FN rate and misclassified more non-toxic proteins as toxic. However, the misclassified sequences may result in hazardous effects, as toxic proteins are a part of virulence factors, and the model may have predicted some of the sequences to be toxic.

### 3.3. Testing Model in Random Protein Sequences Labeled from In Vitro Results

The in vitro toxicity experiments revealed that all four species cause hazardous effects. From this, we presumed that a substantial number of proteins that can be labeled as toxic would be found in the collected protein dataset. Table 7 presents the different prediction results derived from the model trained on the BTXpred data and combined data. Both models were able to identify possible toxic proteins in each species. The number of possible toxic sequences for *M. luteus*, *S. epidermidis*, and *B. subtilis* were almost the same for both models, whereas the model trained using the combined dataset could identify more sequences as toxic for *S. aureus*. We suggest this is because the model trained with the combined dataset has more complex knowledge of possible virulence factors than the model trained with BTXpred data.

## 4. Conclusions

In this study, we proposed the use of ProtBert for the prediction of toxic bacterial proteins. We tested our model on two public datasets and showed that it yields similar results as previous methods for animal toxic protein prediction and toxic bacterial protein prediction. We also trained the model using bacterial virulence factors to further investigate whether the model performance would improve when trained with much broader data. The results showed that our model could correctly classify toxic bacterial protein sequences. The in vitro experiments on unlabeled protein sequences revealed the possibility of finding new toxic protein sequences, and that the in silico method can capture possible toxic protein sequences.

It is noteworthy, however, that even though we could identify possible toxic proteins that may act as virulence factors, we can only presume that these proteins are responsible for hazardous reactions in the in vitro experiments. Hence, we intend to further investigate the link between the identified protein sequences and virulence data through more thorough in vitro experiments.

To strengthen the performance of the in silico protein toxicity prediction, we hope to add other features to the training of the model, such as evolutionary and protein chemical compositions, which are known to create harmful effects.

## Figures and Tables

**Table 1:** Overview of the datasets used.

Dataset	Purpose	Positive	Negative
ToxDL [13]	Training set	4413	5671
	Validation set	59	670
BTXpred [20]	Training set	140	402
	Validation set	43	92
Combined [21,22]	Training set	20,229	14,258
	Validation set	5059	3563
UniprotKB	Test set	216	1000

**Table 2:** Number of collected proteins sequences of each species.

Bacteria Species	Number of Proteins
Staphylococcus aureus	1497
Miccroccus luteus	236
Staphylococcus epidermidis	27
Bacillus subtilis	344

**Table 3:** Test results on the toxic animal protein dataset.

Method	F1-Score	MCC	auROC	auPRC
BLAST ^1^ [30]	0.800	0.801	-	-
BLAST-score ^1^ [30]	0.789	0.775	0.868	0.818
InterProScan ^1^ [31]	0.347	0.402	-	-
Hmmsearch ^1^ [32]	0.185	0.307	-	-
ClanTox ^1^ [11]	0.620	0.604	0.903	0.612
ToxinPred-RF ^1^ [10]	0.667	0.638	0.948	0.716
ToxinPred-SVM ^1^ [10]	0.677	0.648	0.939	0.712
ToxDL ^1^ [13]	0.809	0.793	0.989	0.913
ToxIBTL ^2^ [14]	0.830	0.816	0.953	0.847
This study	0.833	0.818	0.915	0.814

^1^ Results provided from the research in [13]. ^2^ Results provided from the research in [14].

**Table 4:** Test results on the toxic bacteria protein dataset.

Method	Accuracy	F1	MCC	auROC	auPRC
BTXpred ^1^ [20]	96.07%	-	0.9293	-	-
TOXIFY [12]	83.35%	-	-	-	-
This study	97.98%	0.9579	0.9434	0.9671	0.9647

^1^ Results provided from the research in [19].

**Table 5:** Test results on the combined VFDB and toxinpred2 dataset.

Method	F1	MCC	auROC	auPRC
This study	0.9527	0.8845	0.9645	0.9408

**Table 6:** Test data results.

Dataset Used for Training	F1	MCC	auPRC	auROC
BTXpred [20]	0.7790	0.7617	0.8401	0.8239
Combined [21,22]	0.7036	0.6412	0.7388	0.8644

**Table 7:** Test data results on two models trained using BTXpred data and the combined data of VFDB and toxinpred2.

Dataset Used for Training	Staphylococcus Aureus	Micrococcus Luteus	Staphylococcus Epidermidis	Bacillus Subtilis
BTXpred [20]	45	43	5	21
Combined [21,22]	263	49	5	27

## Data Availability

Not applicable.
